# DaT-SPECT assessment depicts dopamine depletion among asymptomatic G2019S LRRK2 mutation carriers

**DOI:** 10.1371/journal.pone.0175424

**Published:** 2017-04-13

**Authors:** Moran Artzi, Einat Even-Sapir, Hedva Lerman Shacham, Avner Thaler, Avi Orr Urterger, Susan Bressman, Karen Marder, Talma Hendler, Nir Giladi, Dafna Ben Bashat, Anat Mirelman

**Affiliations:** 1 Functional Brain Center, The Wohl Institute for Advanced Imaging, Tel Aviv Sourasky Medical Center, Tel Aviv, Israel; 2 Sackler School of Medicine, Tel Aviv University, Tel Aviv, Israel; 3 Department of Nuclear Medicine, Tel-Aviv Sourasky Medical Center, Tel Aviv, Israel; 4 Movement Disorders Unit, Neurological Institute, Tel Aviv Sourasky Medical Center, Tel Aviv, Israel; 5 Genetics Institute, Tel Aviv Sourasky Medical Center, Tel Aviv, Israel; 6 Columbia University, Columbia University Medical Center, New-York, New York, United States of America; 7 Mount Sinai-Beth Israel Medical Center, New York, New York, United States of America; 8 Sagol School of Neuroscience, Tel Aviv University, Tel Aviv, Israel; 9 Department of Psychology, Tel Aviv University, Tel Aviv, Israel; 10 Laboratory for Early Markers of Neurodegenertion, Neurology Institute, Tel Aviv Sourasky Medical Center, Tel Aviv, Israel; Universita degli Studi di Padova, ITALY

## Abstract

Identification of early changes in Dopamine-Transporter (DaT) SPECT imaging expected in the prodromal phase of Parkinson’s disease (PD), are usually overlooked. Carriers of the G2019S *LRRK2* mutation are known to be at high risk for developing PD, compared to non-carriers. In this work we aimed to study early changes in Dopamine uptake in non-manifesting PD carriers (NMC) of the G2019S *LRRK2* mutation using quantitative DaT-SPECT analysis and to examine the potential for early prediction of PD. Eighty Ashkenazi-Jewish subjects were included in this study: eighteen patients with PD; thirty-one NMC and thirty-one non-manifesting non-carriers (NMNC). All subjects underwent a through clinical assessment including evaluation of motor, olfactory, affective and non-motor symptoms and DaT-SPECT imaging. A population based DaT-SPECT template was created based on the NMNC cohort, and data driven volumes-of-interest (VOIs) were defined. Comparisons between groups were performed based on VOIs and voxel-wise analysis. The striatum area of all three cohorts was segmented into four VOIs, corresponding to the right/left dorsal and ventral striatum. Significant differences in clinical measures were found between patients with PD and non-manifesting subjects with no differences between NMC and NMNC. Significantly lower uptake (*p*<0.001) was detected in the right and left dorsal striatum in the PD group (2.2±0.3, 2.3±0.4) compared to the NMC (4.2±0.6, 4.3±0.5) and NMNC (4.5±0.6, 4.6±0.6), and significantly (*p* = 0.05) lower uptake in the right dorsal striatum in the NMC group compared to NMNC. Converging results were obtained using voxel-wise analysis. Two NMC participants, who later phenoconverted into PD, demonstrated reduced uptake mainly in the dorsal striatum. No significant correlations were found between the DaT-SPECT uptake in the different VOIs and clinical and behavioral assessments in the non-manifesting groups. This study shows the clinical value of quantitative assessment of DaT-SPECT imaging and the potential for predicting PD by detection of dopamine depletion, already at the pre-symptomatic stage.

**Clinical registration numbers**: NCT01089270 and NCT01089283.

## Introduction

Parkinson's disease (PD), affects 1% of the population over 60, with incidence rates of 0.3 per 1000 person years in persons aged 55 to 65 [1, 2]. PD pathology is characterized by the accumulation of alpha-synuclein into inclusions in neurons and insufficient formation and activity of dopamine produced within the basal ganglia. When the neurodegenerative process a critical point, when approximately 50–60% of the substantia nigra (SN) neurons are lost and 60–80% of the dopamine content of the striatum is depleted [3, 4], motor symptoms start to immerge and the disease is clinically diagnosed. During this long prodromal pathological process, some individuals may present with non-motor symptoms and signs but these are usually unspecific [5–7]. Although currently there is no established treatment available to alter the underlying neurodegenerative process, there is a global effort to develop disease modifying therapies which will be introduced in the future in the prodromal phase [8].

Recent evidence suggests that PD is caused by a combination of complex genetic and environmental factors. The G2019S mutation in the leucine-rich repeat kinase 2 (*LRRK2*) gene, represents the most common pathogenic mutation identified in PD worldwide, accounting for up to 1–6% of sporadic and 3–19% of familial PD with even higher frequencies in Ashkenazi Jews (AJ) (16% in sporadic and 30% if familial patients) [9–11]. Non-manifesting carriers (NMC) are considered to have an increased risk for future development of the disease [2, 12]. Current estimations regarding G2019S penetrance range between 30–80% at age 80 [13, 14]. Yet, at present there are no sensitive methods to identify those likely to develop the disease.

While the diagnosis for PD is based on motor symptoms, neuroimaging modalities including trans-cranial sonography, MRI and imaging using specific single photon and positron emitting tracers with SPECT and PET technology, are all used for PD assessment and to rule out other disorders [15, 16]. Several imaging studies have been performed on non-manifesting carriers (NMC) with non-converging results reported using MRI, relating to differences in gray matter volume and diffusion tensor imaging (DTI) parameters [17, 18].

DaT-SPECT with dopamine transporter (DaT) tracer is a well-established method for the assessment and investigation of PD by imaging presynaptic dopaminergic function within the basal ganglia, supporting impairment of the dopaminergic networks [15, 19]. DaT-SPECT scan was approved by the European Medicines Agency and by the Food and Drug Administration for in vivo diagnosis for subjects suspected with PD [20]. Asymmetric rostral-caudal decrease in tracer uptake, maximally affecting the posterior dorsal striatum, has been identified in patients with early PD [15, 19, 21]. One preliminary work using PET in seven NMC compared to non-manifesting non-carriers (NMNC), did not find any between group differences [22]. However a recent study detected uptake differences between NMC and NMNC in subjects with R1441G mutation in the LRRK2 gene [23]. It has been suggested that diagnostic accuracy in DaT-SPECT scans might be highly dependent on the user/reviewer’s experience as currently interpretation is mainly visual and therefore semiquantative and subjective. This is particularly relevant if changes are subtle as expected early in the course of the disease. Generation of an objective quantitative automatic or semi-automatic tool for assessment of DaT-SPECT data was the scope of a few publications using atlas-based volumes of interest (VOIs) [24] and by automatic calculation of the striatal uptake relative to background [25].

Thus, in the present study, we aimed to explore potential quantitative differences in DaT-SPECT uptake in patients with PD, NMC and NMNC. We hypothesized that using an objective quantitative tool, based on clinical data would enable detection of subtle changes in NMC, potentially enabling an imaging biomarker for early diagnosis of PD already at the pre-symptomatic stage of the disease.

## Material and methods

### Participants

DaT-SPECT scans and clinical and behavioral assessment were performed on eighty AJ participant, between April 2010 and February 2015. Subjects' characteristics are given in Table 1. Subjects were part of a prospective observational study aimed to assess the genetic basis of PD in AJ performed at the Tel-Aviv Medical Center (TLVMC) as part of a multinational consortium funded by the Michael J Fox foundation. Patients with PD (n = 18, 9 females, mean age 63±9 years, disease duration: 2.41±1.84 years) were included in this study if they were diagnosed with PD by a movement disorder specialist based on the UK Brain Bank diagnosis criteria [26]. Patients were excluded if they had significant psychiatric impairments, used dopamine depletion medications or had additional neurological conditions other than PD.

**Table 1:** Subjects’ characteristics.

	PD (n = 18)	NMC (n = 31)	NMNC (n = 31)	P value
Age	63±9*	48±11	47±12	<0.001*
Gender (M/F)	9/9	14/17	11/20	ns
UPDRS III	16.4±6.9	2.0±2.3	2.2±4.4	<0.001*
MoCA	26.5±2.0	27.5±2.5	27.1±2.2	ns
UPSIT	23.4±3.3*	30.6±3.1	32.2±3.1	<0.001*
BDI	5.2±4.6	3.7±5.2	6.1±9.0	ns
SCOPA AUT	16.7±14.0*	6.2±6.0	7.9±7.8	<0.001*
ESS	7.9±5.0	6.1±2.9	8.0±3.5	ns
RBDQ	3.5±3.3	1.8±1.5	2.4±2.0	ns
NMS	8.8±5.4*	3.7±4.2	4.5±4.3	<0.001*

PD-Parkinson's Disease; NMC- non-manifesting carriers; NMNC- non-manifesting non-carriers; UPDRS III-Unified Parkinson’s disease Rating Scale motor part III; MoCA-Montreal Cognitive Assessment; UPSIT- University of Pennsylvania Smell Identification Test, BDI- Beck Depression Inventory; ns-not significant. SCOPA AUT- Scales for Outcomes in Parkinson’s Disease—Autonomic; ESS—Epworth Sleepiness Scale; RBDQ- REM sleep behavior disorder questionnaire; NMS- non-motor symptoms questionnaire;

*Significant difference from the other groups.

A total of 62 non-manifesting subjects who underwent DaT-SPECT scans were included in this study: 31 non-manifesting non-carriers (NMNC), first degree relatives of patients with the G2019S *LRRK2* gene (20 females, mean age 47±12 years) and 31 NMC (17 females mean age 48±11 years). Carrier status was determined based on an examination of the 6055G_A (G2019S) mutation in exon 41 of the *LRRK2* gene [27, 28]. Participants were unaware of their genetic status during recruitment and scanning. Inclusion criteria for both groups included no overt signs of PD (Unified Parkinson's Disease Rating Scale (UPDRS) part III [motor part] cutoff score = 4) [29], and no significant cognitive impairment (MOCA, cutoff score = 23) [30]. Subjects were excluded if they had a history of significant head trauma, significant neurological disease including overt stroke, were treated with medications for PD or with dopamine depleting medication or were carriers of a *GBA* mutation. The study was approved by the Tel Aviv Medical Center Institutional Review Board committee and all participants provided written informed consent prior to participation (clinical registration numbers: NCT01089270 and NCT01089283).

#### Clinical and behavioral assessments

Clinical assessment included: *Neurological assessment*: based on the Unified Parkinson's Disease Rating Scale (UPDRS) part III assessing disease symptoms and severity; *Cognitive assessment*: the Montreal Cognitive Assessment (MoCA) test, used to assess global cognitive function; *Non-motor assessment*: Scales for Outcomes in Parkinson’s Disease—Autonomic (SCOPA-AUT) [31]; The Beck Depression Inventory (BDI), used to assess mood and depression; the University of Pennsylvania Smell Identification Test (UPSIT), used to assess olfaction; the Epworth Sleepiness Scale (ESS) [32] and REM sleep behavior disorder questionnaire (RBDQ) [33], used to assess sleep. Non motor symptoms were assessed using the Non-Motor Symptoms questionnaire (NMS) [34].

### DaT-SPECT imaging

Before the tracer injection subjects received stable iodine per os (7–10 drops of saturated solution of potassium iodide) to reduce uptake and radiation exposure of the thyroid gland. Then 5mCi (185MBq) of DaTTM were IV injected. Single Photon Emission Tomography (SPECT) acquisition was initiated 3 hours post injection using the Infinia camera (GE Healthcare) with fan beam collimator. Acquisition protocol was 128*128 matrix size and 20 seconds per frame. Data was reconstructed as following ordered subset expectation maximization (OSEM) with 2 iterations and 10 subsets, attenuation correction with coefficient 0.11 and butterworth 0.5 filtering with critical frequency of 0.5 and power 10 and no scatter corrections.

### Analysis of the DaT-SPECT data

#### Data preprocessing

Included, *Realignment* of the DaT-SPECT to 18F-DOPA PET SPECT template using rigid-body transformation and s*patial normalization*, performed using SPM8 (MATLAB 2014b, The MathWorks Inc); *Intensity normalization*; performed using FMRIB Software Library, (FSL) [35] relative to the occipital lobe which served as a reference area. The reference area was defined in each subject based on the MNI structural atlas, (part of FSL), and standardized values *X*_*SDi*_ were calculated as follows:

$$X_{SDi}=\frac{\left[X_{i}-\;\bar{X}\right]}{\sigma_{\bar{X}}}$$

Where *X*_*i*_ = value in voxel *i*; $\bar{X}$ = mean value at the reference area; and $\sigma_{\bar{X}}$ = standard deviation value at the reference area.

#### Generating population-based template of the normal brain

A study-specific probabilistic template was created for the DaT-SPECT data by averaging all standardized maps obtained from the NMNC group (n = 31). The template included only voxels within three standard deviations above the uptake of the background signal, resulting in the striatum area. The obtained template was further used for group comparison analyses.

#### Automatic definition of the volumes of interest

Unsupervised classification of the entire DaT-SPECT data (n = 80) within the striatum area, was performed in order to define VOIs sensitive and specific to PD pathology. The striatum region (from both hemispheres) was segmented into several clusters {*k* = 1–7} using Matlab *k*-means classifier. Optimization of the *k* number was performed using Matlab Silhouette function, with the optimal *k* number selected as the number resulting in the highest similarity for each data point to its own cluster, compared to points in other clusters.

#### Group comparisons of DaT-SPECT uptake

Two analyses were performed in order to identify areas with significant differences in tracer uptake between the three groups (patients with PD, NMC and NMNC):

*Voxel -wise analysis*: voxel-wise analysis was performed on the normalized DaT-SPECT images, within the striatum area, using a permutation-based inference tool for nonparametric statistical thresholding (randomize program, part of FSL). A threshold-free cluster enhancement (TFCE) option was used, including correction for multiple comparisons and adding age as a covariant.

*Volumes of interest analysis*: Mean values of DaT-SPECT uptake within the obtained VOIs were compared between groups.

### Statistical analysis

Univariate analyses, including correction for age and Bonferroni correction for multiple comparisons, were performed using SPSS (SPSS V20, Chicago, IL, USA) to assess differences between groups, for the clinical and behavioral assessments and DaT-SPECT uptake within the four VOIs.

One-sample Kolmogorov-Smirnov test was used to check the distribution of each parameter. Spearman or Pearson correlation were used to assess the association between clinical and behavioral parameters and DaT-SPECT uptake within the four VOIs. Results were considered significant when *p*<0.01.

## Results

Patients with PD were significantly older than the non-manifesting subjects (*p*<0.001), therefore all analyses were adjusted for age. All clinical measures of the non-manifesting groups (NMNC and NMC) were within normal range, reflecting no clinical signs of disease (Table 1). Differences were observed between patients with PD and non-manifesting subjects in motor symptoms as assessed by part III of the UPDRS (*p*<0.0001), olfactory functions as assessed by the UPSIT (*p*<0.0001), and in SCOPA-AUT and NMS (*p*<0.05). No differences in any of the clinical and behavioral parameters, both motor and non-motor, were observed between NMNC and NMC.

### Population-based template of the normal brain

**Fig 1:**
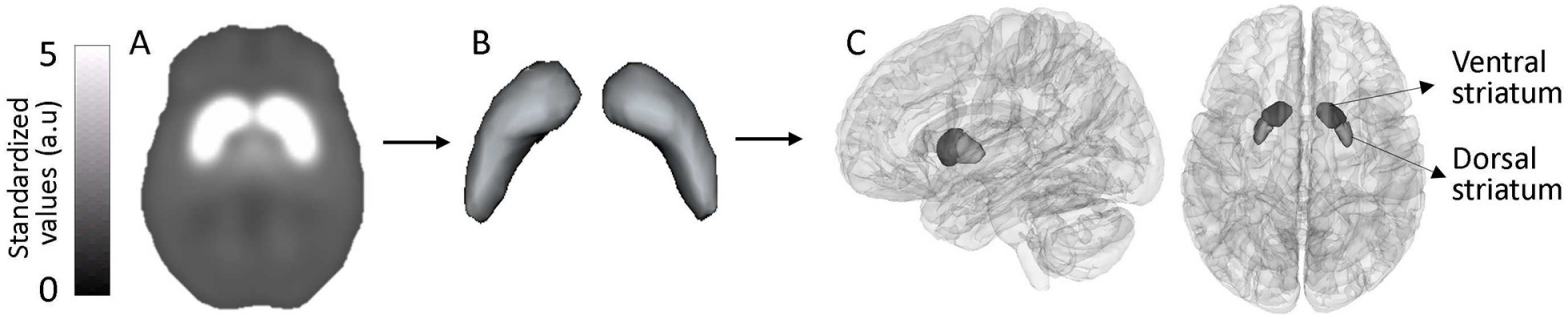
Population based template. (A) Population based template obtained from non-manifesting non carriers of the *LRRK2* mutations (n = 31) group. (B) The defined striatum area. (C) The four defined VOIs: ventral and dorsal striatum in the right and left hemispheres, following *k*-means segmentation of the DaT-SPECT images of the three groups.

Fig 1a shows the population based template obtained from the NMNC (n = 31) group. Only voxels within three standard deviations above the uptake of the background signal were included, resulting in the striatum area (Fig 1b). This area was used for further analysis.

### Definition of the volumes of interest

In order to define the area sensitive to DaT-SPECT uptake in PD, a *k*-means classifier was used to segment the striatum areas aiming to have high homogeneity within segments and good differentiation between segments. The segmentation to VOIs, was performed based on the normalized DaT-SPECT images of all three groups, in order to capture differences between groups and thus obtained VOIs specific to PD. The optimal *k* was found to be 2. Each striatum area (left and right) was automatically segmented into two VOIs, resulting in four VOIs: the right and left ventral and right and left dorsal striatum areas. Fig 1c shows the four VOIs (segments). The volumes of each segment in each hemisphere were: right-hemisphere: ventral striatum = 1.06cc; dorsal striatum = 2.36cc; left-hemisphere: ventral striatum = 1.04cc and dorsal striatum = 2.25cc.

### Differences in DaT-SPECT uptake between groups within VOIs

**Fig 2:**
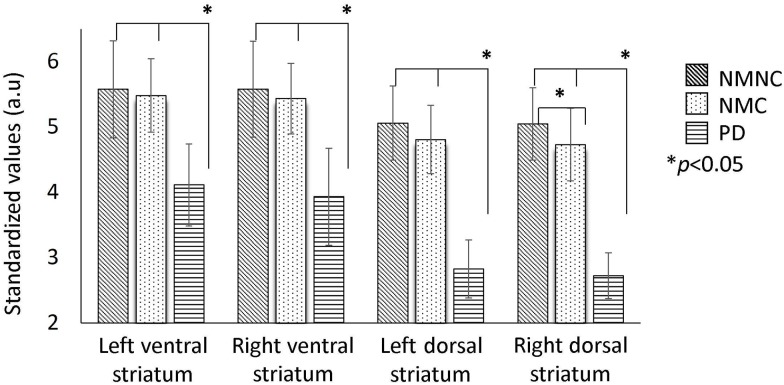
Differences in DaT-SPECT uptake between groups within VOIs. Mean standardized DaT-SPECT values obtained in the striatum VOIs in the three groups. NMNC = non-manifesting non-carriers of *LRRK2* mutations, NMC = non-manifesting *LRRK2* mutations carriers; (*significant group differences, *p*<0.05, corrected for age and for multiple comparisons).

Fig 2 shows mean DaT-SPECT uptake of the three groups, within the four obtained VOIs. Between- group comparisons showed significantly lower uptake in the PD group relative to the other two groups in all four VOIs, dorsal and ventral striatum areas in both hemispheres. In addition, significantly lower uptake was detected in the NMC group relative to the NMNC group in the right dorsal striatum (Fig 2).

### Differences in DaT-SPECT uptake between groups –voxel-wise analysis

**Fig 3:**
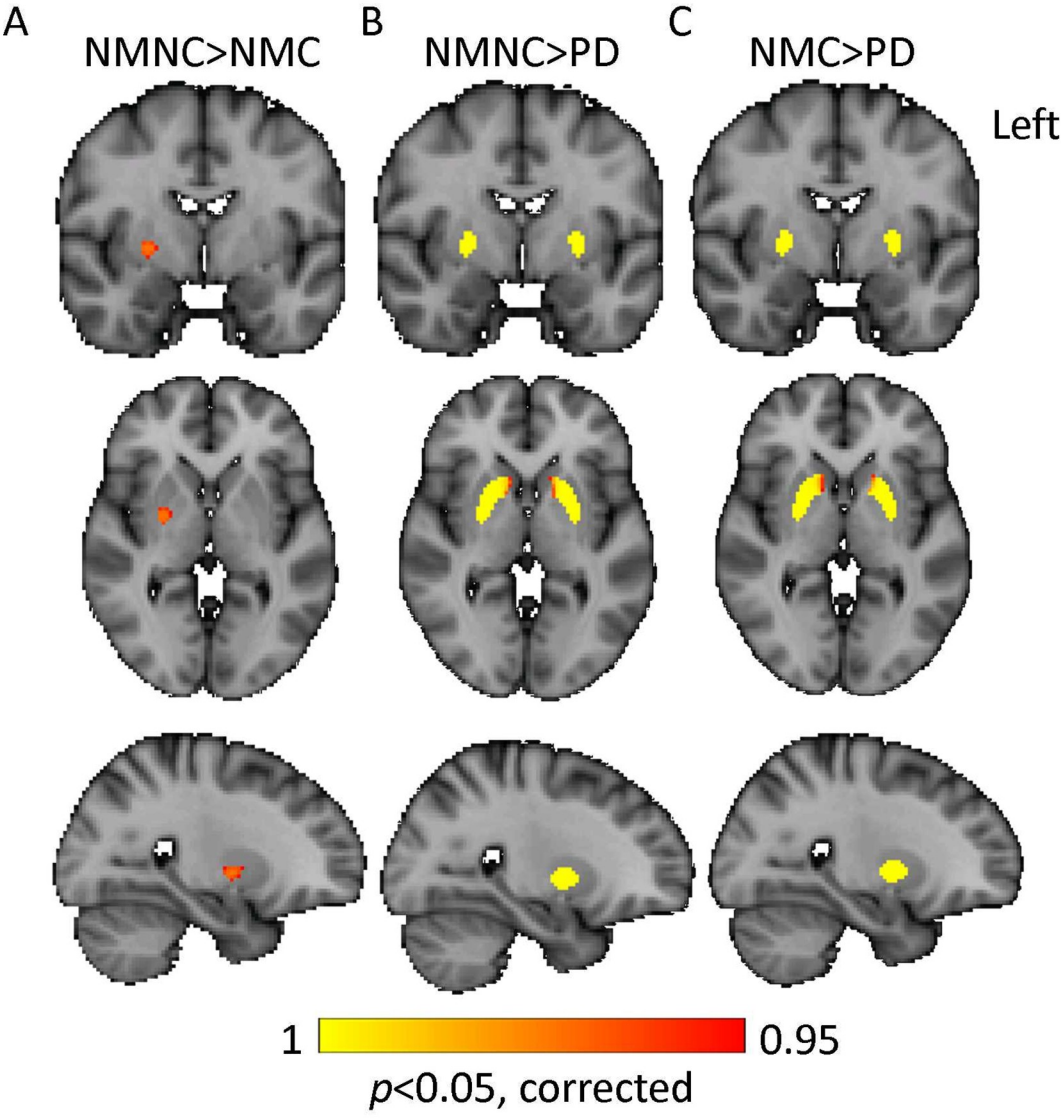
Differences in DaT-SPECT uptake between groups -voxel-wise analysis. NMNC> NMC (A), NMNC>PD (B), NMC>PD (C); NMNC = non-carriers of *LRRK2* mutations, NMC = non-manifesting *LRRK2* mutations carriers.

In order to support our findings from the VOI analysis, voxel-wise comparisons between groups were performed. Results are presented in Table 2 and illustrated in Fig 3. Significantly lower tracer uptake in the left and right dorsal and ventral striatum areas were detected in patients with PD compared to the NMC and NMNC (Fig 2a and 2b). In addition, lower uptake in the right dorsal striatum was detected in the NMC group, compared to NMNC (Fig 2c).

**Table 2:** DaT-SPECT voxel-wise analysis.

Contrast	Structure	MNI (x, y, z)	T value	*p* value FDR corrected	Cluster size (mm^3^)
NMC>PD	Right striatum	20 6 2	5.35	<0.001	3.05
	Left striatum	-20 6 2	6.00	<0.001	2.61
NMNC>PD	Right striatum	20 6 2	4.96	<0.001	2.88
	Left striatum	-20 6 2	5.58	<0.001	2.51
NMNC>MN	Right striatum	26 -4 -0	2.84	<0.001	0.31

PD = patients with Parkinson's disease (n = 18); NMC = non-manifesting LRRK2 mutations carriers (n = 31); NMNC = non-manifesting non-carriers of LRRK2 mutations (n = 31)

### Correlations with behavioral measures

No significant correlations were detected between DaT-SPECT uptake in the four VOIs and any of the clinical and behavioral measures, among non-manifesting participants.

#### Preliminary results—Prediction of disease onset

**Fig 4:**
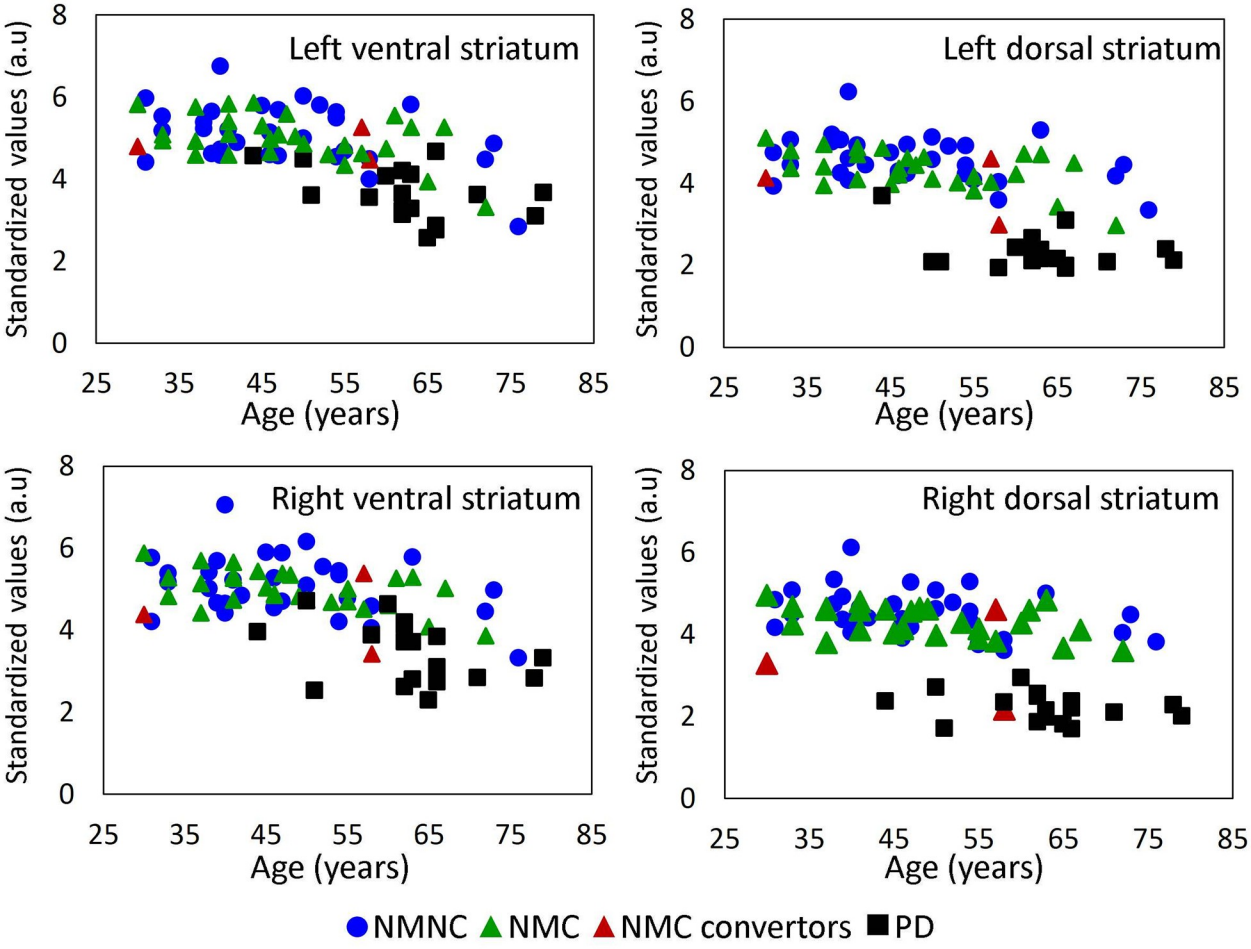
Scatter plots of DaT-SPECT uptake. Scatter plot of the striatum VOI's; Mean standardized values of DaT-SPECT uptake versus age. NMNC = non-manifesting *LRRK2* mutation carriers (blue triangle); NMC = non manifesting non-carriers of *LRRK2* mutation (green triangle); PD = Parkinson patients (black square). NMC who converted to PD patients are marked with red triangles.

Three NMC were clinically diagnosed with PD during a follow up assessment ~24 months after participating in the study. Diagnosis was confirmed by a movement disorders specialist based on neurological examination and the change in UPDRS score. UPDRS III scores at baseline were: 3, 1 and 2 compared to 9, 6 and 9, at 24 months, respectively. Fig 4 shows scatter plots of DaT-SPECT uptake in the four VOIs according to age, of the three groups, with the three phenoconvertors marked with red triangles. Two subjects demonstrated significant lower uptake values in three segments: right and left dorsal and right ventral striatum during their initial assessment (i.e. while still non-manifesting). The third patient, whose uptake values were normal, was re-scanned after a clinical diagnosis of PD was issued, and although he demonstrated reduced uptake with an average of 7% in all four segments relative to the first scan, all values at both time points were within the normal range of general population, showing no evidence for dopaminergic deficit (SWEDDs).

## Discussion

In this study we used quantitative analysis of DaT-SPECT imaging to assess dopamine depletion in patients with PD and in non-manifesting G2019S mutation carriers of the *LRRK2* gene. Our findings confirm the recognized pattern of reduced DaT-SPECT uptake in PD patients, yet demonstrated a reduction in the entire striatum, not limited only to the dorsal regions. In addition, we were able to detect reduced DaT-SPECT uptake in the striatum of NMC as compared to NMNC at a lower magnitude compared to that of patients with PD. Converging results were obtained using voxel-wise and VOI analysis, in line with previous findings [23, 36, 37]. Preliminary results in three subjects demonstrated the potential use of DaT-SPECT imaging for early detection of the disease during the non-manifesting stage in subjects at risk.

A template of DaT-SPECT scan uptake was created based on DaT-SPECT uptake obtained in healthy subjects. Previous studies used different procedures of registration and normalization of DaT-SPECT data to create templates, and used manual definition or atlas based anatomical VOIs to study differences in uptake between groups [38, 39]. In this study, following normalization, the template was created with standard deviation values, relative to the background signal. The VOIs were defined automatically using k-mean segmentation, based on data of DaT-SPECT uptake obtained from all subjects: patients with PD, subjects at risk (NMC) and healthy controls (NMNC). This approach does not rely on morphological or anatomical information in order to identify the striatum area, but is driven by PD pathology itself. Obtaining reference values in each group enabled us to provide patient-specific analysis rather than group analysis, which is crucial when used in clinical evaluation.

Converging results were obtained using voxel-wise and VOI analyses. In routine clinical practice, relative visual reduction in uptake at the dorsal striatum is considered positive for disease. However, using quantitative analysis, reduced uptake in the PD group was detected in *all* four segments, with a larger magnitude in dorsal regions. Reduced uptake was detected in NMC compared to NMNC, being more pronounced in the right dorsal striatum, which is in line with the known mode of progression of the disease, with the dorsal regions of the striatum being affected first [3]. Our findings are also in line with previous studies that detected reduced DaT-SPECT uptake in unaffected subjects at high risk of developing PD [23] [36] [37], suggesting the use of an automatic tool to obtain quantitative values. In two NMC subjects, who later developed motor symptoms, and were diagnosed with PD two years after DaT-SPECT imaging, early diagnosis could have been performed based on reduced uptake in the right and left dorsal striatum. Regarding the third subject who was later diagnosed with PD, previous studies showed that in approximately 10% of patients diagnosed clinically with early PD, DaT-SPECT scans show no evidence for dopaminergic deficit (SWEDDs) [40]. This may explain the results of the third converter who had normal values before and after disease onset, yet presented with clinical symptoms of PD.

We did not find any correlations within the non-manifesting groups between the uptake values and any clinical or behavioral parameters including motor, olfactory, affective and non-motor symptoms, indicating that these parameters were either not sensitive enough or were not directly related to dopaminergic uptake in the striatum. A recent study suggested criteria and probability methodology for the diagnosis of prodromal PD [41]. Adding additional parameters to the probability model, (even in the absence of significant differences compared to healthy subjects) may improve this ability to predict PD especially in high-risk populations.

Penetrance estimations of *LRRK2* G2019S vary widely (24–80%) [42–44] and may depend on ascertainment, ethnic group, gender, and other genetic or environmental modifiers. Our group recently reported a lower than expected penetrance of only 25% at age 80 in a kin-cohort analysis [45]. Thus, the present findings could be related to the presence of the G2019S reflecting an endophenotype, with relatively few NMC eventually developing clinical PD. Because of the relatively low level of penetrance, a cross sectional assessment of NMC is expected to include subjects that will and will not develop PD with subjects in different stages of the pre-motor disease. Therefore differences between NMC and NMNC might be diluted. In this regard, the findings from the two convertors strengthen our methods and suggest the potential predictive value of the analysis for detecting prodromal disease markers.

Several limitations of this study should be considered: there were differences in age between PD and first degree relatives, thus some results may not be detected when controlling for age. In addition, there was a relatively small sample size, and the NMC group included subjects who will eventually convert to PD and those who will not. Thus, grouping them together to detect differences compared to NMNC may obscure some of the results.

In conclusion, quantitative analysis of DaT-SPECT revealed reduced dopamine uptake in the entire striatum in patients with PD compared to healthy controls, not limited to the dorsal regions. In addition, preliminary results among asymptomatic G2019S *LRRK2* MC demonstrated subtle tracer uptake reduction already at the pre-symptomatic stage.
